# The Use of Social Networking Sites for Public Health Practice and Research: A Systematic Review

**DOI:** 10.2196/jmir.2679

**Published:** 2014-03-14

**Authors:** Daniel Capurro, Kate Cole, Maria I Echavarría, Jonathan Joe, Tina Neogi, Anne M Turner

**Affiliations:** ^1^Evidence Based Healthcare ProgramDepartment of Internal Medicine, Escuela de MedicinaPontificia Universidad Catolica de ChileSantiagoChile; ^2^Department of Biomedical Informatics and Medical EducationSchool of MedicineUniversity of WashingtonSeattle, WAUnited States; ^3^Department of Global HealthSchool of MedicineUniversity of WashingtonSeattle, WAUnited States; ^4^Department of Family MedicineSchool of MedicineUniversity of WashingtonSeattle, WAUnited States; ^5^Department of Health ServicesSchool of Public HealthUniversity of WashingtonSeattle, WAUnited States

**Keywords:** public health informatics, public health, social network, health communication

## Abstract

**Background:**

Social networking sites (SNSs) have the potential to increase the reach and efficiency of essential public health services, such as surveillance, research, and communication.

**Objective:**

The objective of this study was to conduct a systematic literature review to identify the use of SNSs for public health research and practice and to identify existing knowledge gaps.

**Methods:**

We performed a systematic literature review of articles related to public health and SNSs using PubMed, EMBASE, and CINAHL to search for peer-reviewed publications describing the use of SNSs for public health research and practice. We also conducted manual searches of relevant publications. Each publication was independently reviewed by 2 researchers for inclusion and extracted relevant study data.

**Results:**

A total of 73 articles met our inclusion criteria. Most articles (n=50) were published in the final 2 years covered by our search. In all, 58 articles were in the domain of public health research and 15 were in public health practice. Only 1 study was conducted in a low-income country. Most articles (63/73, 86%) described observational studies involving users or usages of SNSs; only 5 studies involved randomized controlled trials. A large proportion (43/73, 59%) of the identified studies included populations considered hard to reach, such as young individuals, adolescents, and individuals at risk of sexually transmitted diseases or alcohol and substance abuse. Few articles (2/73, 3%) described using the multidirectional communication potential of SNSs to engage study populations.

**Conclusions:**

The number of publications about public health uses for SNSs has been steadily increasing in the past 5 years. With few exceptions, the literature largely consists of observational studies describing users and usages of SNSs regarding topics of public health interest. More studies that fully exploit the communication tools embedded in SNSs and study their potential to produce significant effects in the overall population’s health are needed.

## Introduction

### Overview

Public health has achieved great advances in improving population health over the past century through education, communication, policy development, and risk management. Public health practitioners and researchers have been exploring new information technologies to more effectively and efficiently communicate with, engage, and educate the public. Today, social media offers a range of possibilities for establishing multidirectional communication and interaction, as well as quickly monitoring public sentiment and activity. These new tools have the potential to help public health meet many of its modern challenges and mandates regarding communicating with, educating, engaging, and monitoring a diverse public [1]. We conducted a systematic review of the use of social networking sites (SNSs) in public health practice and research to better understand the use of these technologies for public health purposes.

### Background

An expanding number of people use the Internet in their daily lives, including for accessing health information [2]. Recently, the growth of interactive and dynamic Web applications has allowed the growth of SNSs, such as Facebook and Twitter. In 2011, 65% of Internet users in the United States reported using SNSs; 61% of Americans aged 18 to 30 years reported using an SNS every day and daily usage by Americans aged 50 to 64 years rose from 20% in 2010 to 32% the following year [3]. Facebook is currently the most popular SNS in the world, topping 1 billion active users, with 580 million who engage with the site daily [4]. Twitter, with 500 million users worldwide [5], has gained a reputation as a way to detect and predict events and sentiments by observing users’ posts (tweets) in real time [6]. In addition to these very large SNSs, innumerable smaller SNSs exist serving a range of interests and needs, from LinkedIn for professional networking to PatientsLikeMe, where patients of similar diseases connect to share treatment resources and support. These sites allow users to engage with and shape their Internet content, from creating a network of connections with other users, to posting their own content, and to reacting and adding to content posted by other users. SNSs are a subset of social media. Social media is a broader concept that encompasses sites that allow users to generate and share content, such as blogs, wikis, and content communities, such as YouTube. SNSs are also characterized by user-generated content, but their defining characteristic is the ability to generate direct communication and 2-way interaction between users, thus generating networks of users [7].

Although the increasing popularity of SNSs is clear, there is no single canonical definition for an SNS. A social networking tool or site is generally distinguished by the creation of individual public profiles and multidirectional communication and collaboration, allowing users to connect to one another within the site [8,9]. Public health researchers and practitioners are interested in the use of SNSs because of the quick and inexpensive access to a broad or specific population that they afford and the possibilities for multidirectional communication that they offer [9].

These qualities give SNSs the potential to expand and enhance core public health functions. For instance, research, surveillance, health education, and linking people with health resources are essential public health services [1]. These tasks are traditionally resource-heavy, requiring in-person participant recruitment and sometimes resulting in lag times in detecting or notifying the public of health threats [10,11]. Furthermore, the public health community often has difficulty reaching certain vulnerable populations who may have the greatest need for services [12]. The large user population and immediate nature of SNSs have the potential to increase the reach and efficiency of these core public health services. Some traditionally hard-to-reach populations, such as adolescents, Hispanics, and low-income Americans, use SNSs at a rate higher than the general population [13] providing a new opportunity for inexpensive public health communication to key demographics. The Centers for Disease Control and Prevention (CDC) provides tools for public health departments to use SNSs to extend the reach of their campaigns [14]. Previous publications have examined the use of SNSs for sexual health promotion, dissemination of health information, and recruitment of difficult-to-reach populations, such as individuals who seek sex online [15-17]. Thus, SNSs may provide new opportunities to effectively achieve the aims of public health.

Given the increasing popularity of SNSs, and the range of possibilities that they offer for public health practice and research, we conducted a systematic review to assess the current uses of SNSs for public health practice and research. This review will serve to inform public health practitioners and informatics researchers of the state of knowledge in the field and identify gaps where more research is needed.

## Methods

### Literature Search

We conducted a database search that included PubMed, EMBASE, and CINAHL Plus using a query consisting of an extensive list of names of specific SNSs, as well as the terms “social networking,” “social network site,” and “public health.” Given the constant evolution of SNSs—with new sites being added while others disappear—we utilized a list of 199 specific SNS names from the corresponding Wikipedia entry [18] current up to the search date. We eliminated all SNS titles that did not generate results. This process yielded the following PubMed search query:

(“social networking”[All Fields] OR “social network site”[All Fields] OR twitter[All Fields] OR facebook[All Fields] OR patientslikeme[All Fields] OR myspace[All Fields] OR renren[All Fields] OR kaixin[All Fields] OR whyville[All Fields]) AND (“public health”[All Fields] OR “public health”[MeSH terms]) NOT “behavior, animal”

MeSH terms

The query was modified to fit specific requirements of each of the databases searched. We included articles published up until March 31, 2012. We also conducted a manual search through the references of the articles retrieved through the electronic search.

### Article Selection

Two researchers reviewed every article to determine if inclusion criteria were met. Discrepancies were resolved through discussion. We included articles that met the following inclusion criteria:

1. Published in peer-reviewed journals,
2. Featured SNSs as the primary subject of study or a main component of the study methodology, and
3. Focused on a topic of public health practice or research or a disease of public health importance. We used the CDC’s 10 Essential Public Health Services [1] and the Information Access for the Public Health Workforce’s list of public health disease processes as criteria to determine public health relevance [19].

We excluded narrative reviews and articles that did not constitute original research communications, such as letters to the editor, whitepapers, and comments.

### Data Extraction

After selecting the articles for inclusion, authors extracted data using an online form specifically designed for this purpose. The extracted data included publication year, location (defined by the location on the study population), study design, sample size, purpose of the study, specific SNS involved (ie, Facebook, Twitter), how the SNS was used (ie, recruiting study participants, promoting public health messaging), target population, and public health topic. We also categorized each article according to whether the study described the use of SNSs for activities currently performed by public health practitioners (we labeled this *practice*) or whether it described novel uses related to public health (we labeled this *research*). Because some studies analyzed individual posts on an SNS as opposed to including individuals or SNS page profiles corresponding to a single person, we also captured the unit of analysis. Data was entered into a specially constructed spreadsheet generated for analysis.

During this step, every article was randomly assigned to 2 researchers to assess intercoder reliability and discrepancies were resolved through discussion with other members of the team.

## Results

**Figure 1:**
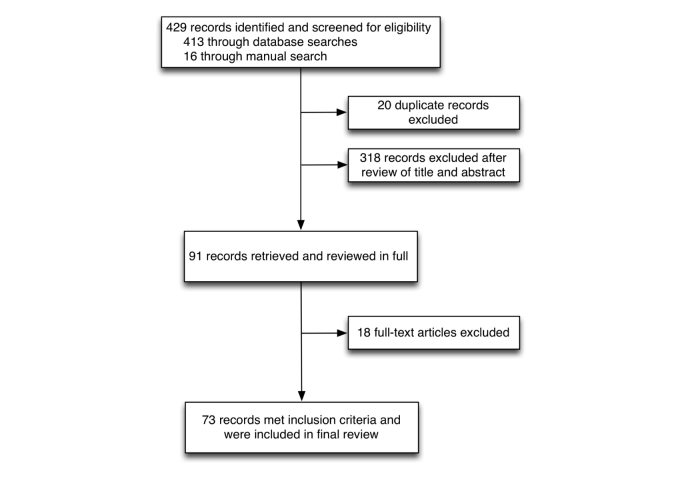
Prisma flow diagram describing the different literature search and selection stages.

We ran our search in July 2012. The literature review retrieved 429 individual articles that were screened for eligibility. A total of 20 duplicates were excluded. After reviewing title and abstract, 318 articles did not meet our inclusion criteria. We reviewed 91 articles in full. Another 18 articles were removed after reviewing the full text because they did not meet our inclusion criteria. Therefore, our final review included 73 unique articles. See Figure 1 for the detailed description of the search process. Table 1 includes a complete description of the included articles.

**Table 1:** Brief description of all included articles about social networking sites (SNSs).

Author/year	Description/general purpose	Social networking site
Moreno 2007 [20]	Determine the prevalence of personal health risk behavior descriptions and identifiable information on MySpace	MySpace
Ybarra 2008 [21]	Determine the use of SNS for unwanted sexual solicitation or Internet harassment of youth	Unrestricted
Blackwell 2009 [22]	Assess the frequency of men who have sex with men (MSM) requesting safer sex when soliciting sex on SNS	SNS targeting MSM
Chou 2009 [23]	Identify the sociodemographic and health-related factors associated with current adult social media users in the US	Unrestricted
Jenssen 2009 [24]	Determine exposure of adolescents to tobacco advertisements on the Internet	Facebook
Moreno 2009 [25]	Determine adolescents’ interpretations of alcohol references displayed on SNS	Unrestricted
Moreno 2009 [26]	Determine the prevalence of and associations among displayed sexual, substance use, and violent risk behavior on teen MySpace profiles	MySpace
Moreno 2009 [27]	Determine whether an online intervention reduces references to sex and substance use on teen SNS profiles	MySpace
Rogers 2009 [28]	Compare differences in emotional self-disclosure between young adults who prefer face-to-face therapy to those who prefer Internet therapy	Facebook
Silenzio 2009 [29]	Explore SNS as a venue for suicide prevention among lesbian, gay, and bisexual young people	MySpace
Yoshimitsu 2009 [30]	Describe the use of a SNS by depressive patients	SNS targeting depressive patients
Bull 2010 [31]	Explore ethics issues with research when using SNS to contact youth	Facebook
Chew 2010 [32]	Evaluate the use of Twitter as an “infodemiology” method during the 2009 H1N1 outbreak	Twitter
Freeman 2010 [33]	Explore whether employees of tobacco companies are promoting tobacco products on Facebook	Facebook
Griffiths 2010 [34]	Examine how young people in New Zealand engage with alcohol and reproduce alcohol marketing messages and alcohol-related branding on Bebo	Bebo
Huang 2010 [35]	Describe the uses of SNS during the natural disaster in Taiwan	Twitter, Plurk
Keelan 2010 [36]	Determine the reaction of the public to the HPV vaccine on social media	MySpace
Kontos 2010 [37]	Describe inequalities in SNS use	Unrestricted
Mitchell 2010 [38]	Describe the variety of ways SNSs are used to facilitate the sexual exploitation of youth, as well as identify victim, offender, and case differences between arrests, with and without a SNS nexus	Unrestricted
Moreno 2010 [20]	Analyze older adolescents’ displayed alcohol references on MySpace	MySpace
Scanfeld 2010 [16]	Categorize tweets mentioning antibiotics and to explore cases of misunderstandings and misuse of antibiotics	Twitter
Stephenson 2010 [39]	Examine the use of an online survey to collect data on the experience and perpetration of intimate partner violence among self-identifying gay and bisexual men in the United States	MySpace
Stulhofer 2010 [40]	Provide a better understanding of the likely mechanisms underlying regular condom use	Multiple
Baptist 2011 [41]	Describe asthma patients’ use of social media and SNS	Unrestricted
Centola 2011 [42]	Analyze how homophily—the tendency of social contacts to be similar to one another—can affect health behavior change in the context of a SNS	Self-created SNS
Chilvers 2011 [43]	Case study of how school nurses began a Facebook page to connect young people with services, schedule appointments, and provide health promotion	Facebook
Dowdell 2011 [44]	Evaluate online social networking patterns among adolescents, young adults, and sexual offenders to provide information to better focus online youth protection education and prevention efforts for nurses and other health care providers	Unrestricted
Feldacker 2011 [45]	Measure the degree to which SNS can increase recruitment for HIV testing	Multiple
Frost 2011 [46]	Describe patient-reported outcomes as a source of evidence in off-label use of drugs within an online community	PatientsLikeMe
Gamage 2011 [47]	Describe effectiveness of Facebook to advertise STI health services and recruit rural young people	Facebook
Gold 2011 [15]	Examine the extent to which SNS are currently being used for sexual health promotion and describe the breadth of these activities	Unrestricted
Grosskopf 2011 [48]	Understand the characteristics, beliefs regarding the risk of HIV infection, and sex-related behaviors of MSM who use SNS to seek sex partners	SNS targeting MSM
Heaivilin 2011 [49]	Analyze tweets to characterize content related to tooth pain	Twitter
Khosropour 2011 [17]	Examine the demographic and behavioral characteristics of MSM enrolled in an online study to determine the factors that predicted retention of participants in an online, prospective follow-up study of MSM	MySpace
Levine 2011 [50]	Describe the process of conducting formative research on MySpace in an effort to involve youth of color in the design of an Internet intervention	MySpace
Liang 2011 [51]	Assess the prevalence of leading pharmaceutical company presence and drug product marketing in online interactive social media technologies	Multiple
Lord 2011 [52]	Facebook survey to gain a better understanding of the beliefs and attitudes that college students have about prescription drug misuse	Facebook
Moreno 2011 [53]	Evaluate depression disclosures by college students on Facebook	Facebook
O’Dea 2011 [54]	Explore SNS as a source of peer mental health support in rural areas	Unrestricted
Pemu 2011 [55]	Determine acceptability and efficacy for diabetes self-management behavior change, and success factors for use of e-HealthyStrides, a SNS designed specifically for diabetes patients	e-healthyStrides
Pletneva 2011 [56]	Present attitudes toward Internet use for health purposes among health care consumers and professionals	Facebook, Twitter
Ridout 2011 [57]	Investigate the presentation of alcohol-identity on SNS	Facebook
Rosselli 2011 [58]	Evaluate an innovative model, “@Prevention,” designed to enhance the culture of prevention among health care professionals and to improve health literacy among the population through information and social marketing	Facebook
Shaw 2011 [59]	Understand the online health information-seeking behaviors of people with diabetes	Unrestricted
Signorini 2011 [60]	Examine the use of Twitter to monitor H1N1 disease activity and public concern	Twitter
Stephenson 2011 [61]	Use Facebook to recruit participants for a study examining reporting of intimate partner violence among MSM in South Africa and associations between intimate partner violence and sexual risk taking	Facebook
Su 2011 [62]	Apply machine intelligence for improving retrieval of health information from Twitter	Twitter
Sullivan 2011 [63]	Determine bias in survey recruitment on MySpace	MySpace
Weitzman 2011 [64]	Test the willingness of an online diabetes community to share data for public health research	TuDiabetes network
Young 2011 [65]	Study the correlation between use of SNS and risky sexual health behavior	Unrestricted
Egan 2011 [66]	Assess the extent to which adolescents display alcohol consumption behaviors on SNS	Facebook
Egan 2011 [67]	Assess the extent to which college students display stress-related emotions on SNS	Facebook
Litt 2011 [68]	Study the role that the social norms displayed on SNS, in the form of profile, play on alcohol-related cognitions in adolescents	Facebook
Selkie 2011 [69]	Understand participants’ willingness to use SNS to obtain information regarding sexual health education	Unrestricted
Chunara 2012 [70]	Assess correlation of volume among cholera-related HealthMap news media reports, Twitter postings, and government cholera cases reported in the first 100 days of the 2010 Haitian cholera outbreak	Twitter and HealthMap
D’Alessandro 2012 [71]	Investigate how college students can be social support catalysts for organ donation and how social and attitudinal dimensions impact organ donor registration, in order to design a SNS organ donation registration campaign	Facebook and YouTube
Gowen 2012 [72]	Examine the ways that young adults with mental illnesses currently use social networking and how they would like to use a social networking site tailored to them	Unrestricted
Jones 2012 [73]	Describe the use of Facebook in the re-recruitment and tracking of adolescent girls in a follow-up study on physical activity	Facebook
Krakower 2012 [74]	Recruit participants from SNS to assess the awareness of, interest in, and experience with pre-exposure prophylaxis (PrEP) among MSM before and after a trial demonstrating PrEP’s effectiveness	SNS targeting MSM
Leighton 2012 [75]	Investigate consumers’ understanding of direct-to-consumer genetic test results using a Facebook survey	Facebook
Lowe 2012 [76]	Investigate social factors, including SNS, that help women who quit smoking because of pregnancy from returning to smoking after giving birth	Unrestricted
Margolis 2012 [77]	Assess HIV testing among adult male members of an MSM sexual networking website, analyze factors associated with “never testing,” and describe reasons for not testing	SNS targeting MSM
Moreno 2012 [78]	Examine association between alcohol use/drinking references on Facebook and reported problem drinking	Facebook
Moreno 2012 [79]	Examine the associations between displayed alcohol use and intoxication/problem drinking references on Facebook and self-reported problem drinking using a clinical scale	Facebook
Norman 2012 [80]	Describe how SNS, interactive blogging, and social media and multimedia platforms have been used to engage youth around issues of health and social action	Unrestricted
Oh 2012 [81]	Test in what ways Korean diabetes patients’ exchanges of computer-mediated social support (CMSS) in diabetes online communities influence their sense of empowerment and intention to actively communicate with their doctor	SNS for diabetes patients
Pantic 2012 [82]	Investigate the relationship between SNS use and depression indicators in adolescents	Unrestricted
Rice 2012 [83]	Use social network analysis to examine the acceptability of a youth-led, hybrid face-to-face and online social networking HIV prevention program for homeless youth	Multiple
Robertson 2012 [84]	Describe the role of SNS and text messaging as sources of contagion and obstacles to recognition in an adolescent suicide cluster	Bebo
Sullivan 2012 [85]	Analyze the online content of concussion-related tweets on Twitter to determine the concept and context of mild traumatic brain injury communication online	Twitter
Thackeray 2012 [86]	Assess the extent to which state health departments are using social media to engage audiences	Unrestricted
Wagenaar 2012 [87]	Compare factors associated with low HIV/AIDS knowledge among Internet-using MSM in South Africa to those in the United States	Facebook
Ramo 2012 [88]	Recruit patients for a tobacco and substance abuse study using an SNS	Facebook

Despite the emergence of Internet SNSs in the late 1990s, the topic did not become prominent in the public health literature until the late 2000s. Since then, the number of publications describing the use of SNSs for public health research and practice has been steadily increasing. In 2007, there was only 1 publication, increasing to 9 in 2009, 12 in 2010, 31 in 2011, and 19 in the first 3 months of 2012. Regarding the location of the study, 43 of the 73 articles (60%) were conducted in the United States. Using the World Bank classification of countries, 14 were conducted in other high-income countries and 1 was conducted in a middle-high income country (South Africa). Only 1 study was conducted exclusively in a low- or low-middle income country (Haiti), but 13 articles explicitly included SNSs and their users without any reference to a particular region or country.

**Figure 2:**
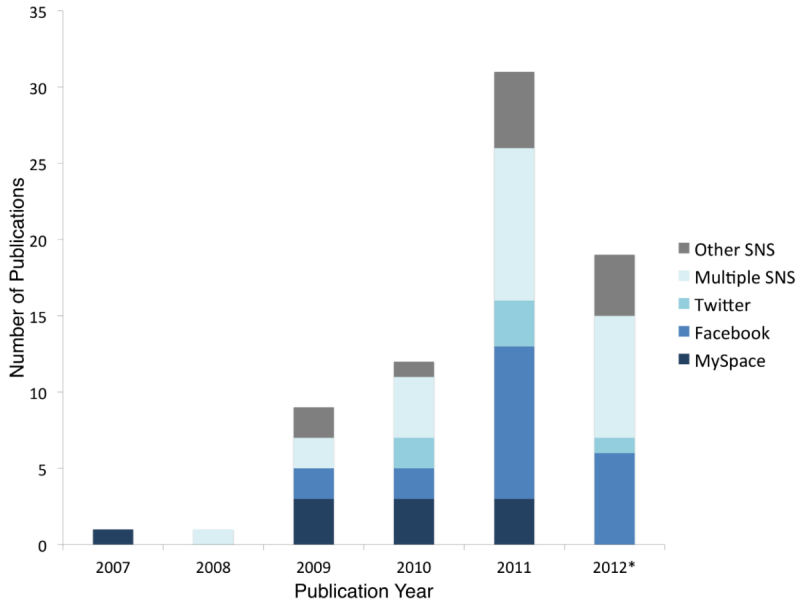
Number of publications and type of social networking sites (SNSs) per year describing the use of SNSs for public health research or practice. *2012 only includes publications through March 31.

Facebook was the most commonly used SNS (27%). In all, 10 of 73 studies (14%) used MySpace, 6 (8%) used Twitter, and 12 (17%) used other SNS, including Bebo, Friendster, LinkedIn, and PatientsLikeMe. There were 25 (34%) that studied multiple SNSs or did not limit by any specific site (Figure 2). Figure 2 shows the changes over time, with MySpace being most frequently used in the early years, and the later appearance of Facebook, Twitter, and other SNSs.

Most studies (63/73, 86%) were cross-sectional observational studies providing descriptions of SNS usage or SNS users. Examples of cross-sectional studies include a study by Jenssen et al [24] analyzing adolescents’ exposure to tobacco advertisements on SNS and a study by Kontos et al [37] in which the authors studied inequalities in SNS usage and its implications for public health communications. Only 5 studies used an experimental design to test a specific intervention, and all 4 were randomized controlled trials. For instance, Centola [42] randomized participants to different user groups on an SNS to study the effect of homophily (similar social contacts) on health behavior change. We found 1 systematic review of non-randomized controlled trials published by Gold et al [15], in which the authors explored the use of SNSs for sexual health promotion.

Articles describing the use of SNSs for public health practice (n=15) focused primarily on hard-to-reach populations. Five articles used SNSs to reach youth and adolescents, or individuals at risk for sexually transmitted diseases (STDs) and human immunodeficiency virus (HIV), to connect them with available services. In an article by Feldacker et al [45], the authors describe their positive experience using SNSs to contact students at high risk for HIV and offer them HIV testing. Another significant use of SNSs for public health practice was to promote healthy behaviors. For example, Pemu et al [55] designed a diabetes management and education SNS and evaluated it with diabetes patients. Other activities routinely conducted by local health departments for which SNSs are being used included disease surveillance and communications during natural disasters [34,59]. See Table 2 for the frequency of SNSs in public health research publications.

**Table 2:** Frequency of uses for social networking sites (SNSs) in public health research publications (n=58).

Use	n (%)
Description of users or usage	39 (67)
Participant recruitment	14 (24)
Outcome assessment	3 (5)
Other	2 (3)

With respect to target population, 32 of 73 studies (44%) focused on young users, such as teenagers, adolescents, or college students. The second largest group of studies targeted the general population without specific restrictions (15/73, 20%). In all, 6 of 73 studies (8%) focused on individuals with specific diseases. Articles focused on a wide array of public health issues; 21 studies (29%) focused on sexual and reproductive health, 17 (23%) on general health promotion strategies, 14 (19%) on noncommunicable diseases, 13 (18%) on alcohol, tobacco, and substance abuse, and 8 (11%) on mental health (see Table 3).

**Table 3:** Frequency of population targeted and public health issue covered in all 73 included articles.

Target population and public health issue		n (%)
**Target population**		
	Youth and young adults	32 (44)
	General population	15 (21)
	Patients with specific chronic noncommunicable diseases	6 (8)
	Patients at high risk for sexual or relationship health diseases	11 (15)
	Other	9 (12)
**Public health issue**		
	Sexual, reproductive health	21 (29)
	General health promotion	17 (23)
	Chronic noncommunicable diseases	14 (19)
	Alcohol, tobacco and substance abuse	13 (18)
	Mental health	8 (11)

## Discussion

### Principal Findings

With over 1 billion active users worldwide on Facebook alone [4], SNSs have become a place where individuals express themselves and interact with one another. Multidirectional communication tools—a hallmark of these sites—allow for rapid and collaborative dissemination of information. This scenario opens up great opportunities for assessing a population’s risk factors and monitoring their health. Thus, SNSs represent a new avenue of action for public health researchers and practitioners. This systematic review provides an overview of the literature concerning the current use of SNSs in public health.

Our results suggest that the application of SNSs to public health research and practice is still maturing. Most articles in this review describe relatively passive approaches to SNSs, rather than harnessing the full potential of SNSs in terms of multidirectional communication and networking. For example, most studies that used SNSs for recruiting study participants simply posted a recruitment ad or survey on the SNS, much as they could on a regular website. In contrast, 2 studies utilized SNSs not only to recruit study participants, but also to maintain contact with participants; thus, improving long-term participation [17,73]. These findings are consistent with the gaps identified in a recently published systematic review. Although different in scope—that review explored the use of social media for health communication—the authors also identified a lack of studies evaluating the impact of such novel technologies [7]. However, examples of more complex uses of SNSs are starting to emerge. Norman and Yip [89] utilized a wide range of SNS capabilities to first recruit young adults to create their own SNS content and then use this content to recruit and communicate with additional young adults around a host of health promotion issues.

Because the popularity of SNSs is a recent phenomenon, we are confident that these formative studies are the required foundation that will provide key knowledge to inform more interactive and experimental studies in the future. The formative study design of these initial SNS studies is suggestive of a developing domain. Most studies utilized a cross-sectional observational design; only 4 were experimental studies and 1 was a systematic review. This preponderance of nonexperimental designs is consistent with what is seen in other nascent domains, such as in the use of information technologies to help treat noncommunicable conditions [90]. It is likely that in coming years we will see an increase in the number of experimental studies using SNSs. For example, one of the studies included in this systematic review was recently followed by a randomized controlled trial that tested the effectiveness of Facebook messages to decrease risky sexual behaviors [91]. In the field of public health research and practice, SNSs may be an attractive low-cost tool to explore and test public health interventions with a large number of diverse participants—interventions that historically have been difficult to conduct using more traditional methods.

One critical—and maybe limiting—issue to be considered when designing randomized trials involving SNSs is their rapid change to potential obsolescence. We were able to show an important number of studies were conducted using MySpace initially, a situation that changed after 2010 in parallel with a decline in the number of MySpace users [92]. These rapid changes in SNS utilization might not be entirely compatible with the time it takes to obtain funding for a randomized trial.

In addition to the types of studies, our findings suggest that although SNSs offer an opportunity to reach a wide range of the population at lower costs when compared to traditional communication methods [93], most published studies have been conducted in high-income countries—predominantly in the United States. Only one study was conducted in South Africa, a higher middle-income country. The cause of this phenomenon is unclear. With the accelerated adoption of mobile broadband and smartphones in both developed and developing countries [94], we will probably begin to see a broader use of SNSs for public health research and practice in low- and middle-income countries in the future.

SNSs provide unique research and practice benefits beyond their low cost, high-reach, multidirectional communication. SNSs appear to be especially well-suited to research and practice on “taboo” public health topics, with nearly half (44%) of the studies included in our review addressing sexual and reproductive health and/or alcohol, tobacco, or substance use. Multiple aspects of SNSs make them suitable for such topics: SNSs afford a high level of anonymity; people display stigmatized behavior more freely online (visiting SNSs for sex, posting about their depression, etc) [95] and youth and young adults, a population largely affected by these issues, has the highest rate of SNS usage of any age group [3]. Not only do SNSs allow easier exploration of traditionally hard-to-discuss topics, they can also facilitate identification of and outreach to certain traditionally hard-to-reach populations, such as men who have sex with men (MSM) with risky sexual behavior [47,76,86], homeless youth [64,82], and young people suffering from mental illness [52,71]. Finally, SNSs allow for the exploration of public health issues associated with the increasing use of SNSs, such as promotions for drugs and tobacco on SNSs [31,50], online harassment and sexual solicitation of youth [20], and depression associated with SNS use [81].

### Study Limitations

The main limitations of this systematic review arise from our search strategy, which included articles only published in scholarly journals, thus excluding communications published in the grey literature. In addition, some articles might have been missed because of limitations of our search terms. We tried to minimize this by including a broad list of SNS keywords in our list of terms.

### Conclusions

An increasing number of studies involving the use of SNSs within the domain of public health have been published and the frequency and complexity of such studies parallels the growing popularity of SNSs in general. Most of the published studies are descriptive and take a limited approach to using SNSs. When the multiple possibilities that emerge from low cost are considered (eg, high visibility, multidirectional communications), it is obvious that there is a gap in range of possible study areas in public health and SNSs. As the field matures and our knowledge concerning the most effective ways to use these technologies to support public health research and practice matures, we expect to see additional innovative uses of SNSs to engage diverse and broad populations with the goal of better understanding and improving health.

## Abbreviations

CDC: Centers for Disease Control and Prevention
HIV: human immunodeficiency virus
MSM: men who have sex with men
SNS: social networking sites
STD: sexually transmitted diseases
